# *In vitro* effect of bergamot (*Citrus bergamia*) juice against *cag*A-positive and-negative clinical isolates of *Helicobacter pylori*

**DOI:** 10.1186/s12906-015-0769-2

**Published:** 2015-07-30

**Authors:** Angela Filocamo, Carlo Bisignano, Nadia Ferlazzo, Santa Cirmi, Giuseppina Mandalari, Michele Navarra

**Affiliations:** Dipartimento di Scienze del Farmaco e Prodotti per la Salute, University of Messina, Viale Annunziata, 98168, Messina, Italy; Dipartimento di Scienze Biologiche e Ambientali, University of Messina, Sal. Sperone 31, 98166 Messina, Italy

**Keywords:** Bergamot, *H. pylori*, Antimicrobial, *Citrus bergamia*, Natural products, Antibiotic resistance, Synergism, Complementary and alternative medicines

## Abstract

**Background:**

*Helicobacter pylori* infection has been associated with chronic gastritis, peptic ulcer and gastric carcinoma as over half of the world's population is colonized with this gram-negative bacterium. Due to the increasing antibiotic resistance, its eradication rates fails in a great portion of patients. A number of studies showed that molecules largely distributed in commonly consumed fruits and vegetables may have antimicrobial activity. The aim of the present study was to investigate the effect of bergamot juice (BJ) against *Helicobacter pylori in vitro*. The potential therapeutic combination between BJ and the antibiotics amoxicillin (AMX), clarithromycin (CLA) and metronidazole (MTZ) has also been evaluated.

**Methods:**

The minimum inhibitory concentration (MIC) of BJ, AMX, CLA and MTZ against 2 ATCC and 32 clinical isolates of *H. pylori* was assayed according to CLSI. The checkerboard method was used to determine the efficacy of the association BJ with the three reference antibiotics.

Killing curves were performed on the two *cag*A-positive ATCC strains of *H. pylori* (ATCC 43504 and ATCC 49503), on the clinical isolate *cag*A-positive HP6 strain of *H. pylori* and on the clinical isolate *cag*A-negative HP61 strain of *H. pylori*.

**Results:**

BJ (2.5 %, v/v) inhibited the growth of 50 % of the *H. pylori* clinical isolates, whereas 5 % (v/v) inhibited 90 %. AMX was the most effective antibiotic against the reference strains and the clinical isolates, followed by CLA and MTZ. In the combination assays, synergism was observed between BJ and AMX and between BJ and MTZ against both the reference strains and the clinical isolates. Indifference was observed between BJ and CLA.

**Conclusions:**

BJ was effective *in vitro* against *H. pylori* and the genotype status of the clinical strains may have an impact on its susceptibility. The synergistic combination of BJ and antibiotics could be used to prevent or treat resistance.

## Background

Infection with *Helicobacter pylori* (*H. pylori*), identified as group 1 carcinogen by the International Agency for Research on Cancer, has been associated with chronic gastritis, peptic ulcer and gastric carcinoma [1, 2]. Its incidence is highly variable in relation to geography, ethnicity, age and socioeconomic factors. Infection rates are similar for men and women and increase progressively with age. The estimated prevalence is 70 % in developing countries, mainly in the young population, and 30 %–40 % in the developed countries [3].

The virulence of the infecting strain is believed to affect the severity of gastroduodenal diseases caused by *H. pylori*. Despite the wide genetic diversity of *H. pylori* involved in its pathogenesis, a number of genetic loci, such as the cytotoxin-associated gene (*cag*A) and the vacuolating cytotoxin gene (*vac*A), have been identified. *Vac*A, present in all *H. pylori* strains, contains two variable parts relevant to virulence [4], whereas c*ag*A, not present in every *H. pylori* strain, is a marker for a pathogenicity island (PAI) [5] associated with more severe clinical outcomes and an increased risk of developing gastric cancer and peptic ulcer [6]. Although heterogeneity in single *cag*A and *vac*A genes in *H. pylori* have been associated to specific clinical effects [7], some studies suggested that genetic heterogeneity is due to geographical diversity and host environment [8].

The standard treatment for *H. pylori* related disease is a combination of antimicrobial agents and anti-acid agents [9]. The first line of treatment regimens consist of a triple therapy, carried out through the recruitment of a proton pump inhibitor (PPI) and two antibiotics, amoxicillin (AMX) and clarithromycin (CLA), or metronidazole (MTZ) and CLA. Quadruple therapy obtained by the association of PPI, bismuth and two antibiotics (AMX + CLA, or MTZ + tetracycline (TET)) is equivalent or little superior in the eradication rates, although reducing the compliance [3]. However, standard PPI-based therapy fails in up to 30 % of patients, and eradication rates were reduced to 70–80 % over the last few years. This is mostly due to increasing antibiotic resistance which favors recurrence of *H. pylori* infection, particularly high in developing countries.

Due to common side effects and the development of antimicrobial resistance [10], a number of natural compounds have been tested as alternatives [11–13]. We have recently demonstrated that polyphenolic fractions from almond skin were active against standard strains and clinical isolates of *H. pylori* [14].

*Citrus bergamia* Risso et Poiteau (bergamot) is an endemic plant of the Calabrian region in Southern Italy, mostly used for the extraction of its essential oil from the fruit peel, much employed in the fragrance industry and lesser exploited in pharmaceutical and food applications. Bergamot juice (BJ), obtained from the endocarp of the fruit, is considered a byproduct barely utilized by the food industry [15]. Recently, we focused on potential beneficial effects of BJ. We demonstrated that BJ inhibited signaling pathways linked to cancer-associated aggressive phenotype, thus reducing proliferation, adhesion and migration both *in vitro* [16] and *in vivo* [17]. In addition, we documented that the antiproliferative effect of BJ was due to its flavonoid fraction (BJe), which inhibited proliferation inducing apoptosis in human colon cancer cells [18]. Moreover, we provided evidence that BJe possess antioxidant effects [19] and inhibited both gene expression and secretion of LPS-induced pro-inflammatory cytokines in a model of LPS-stimulated THP-1 cell line [20]. Moreover, BJe exerted anti-inflammatory effects also in an animal model of experimental colitis [21], suggesting a possible role in treating rheumatoid arthritis with a good balance between efficacy and safety [22]. Earlier studies demonstrated that the polyphenolic fraction of BJ reduced serum cholesterol, triglycerides and glycaemia in patients suffering from metabolic syndrome [23], supporting previous findings obtained in animal models [24].

The aim of the present study was to investigate the antimicrobial properties of BJ against *H. pylori* strains and the interaction between BJ and the antibiotics AMX, CLA and MTZ.

## Methods

### Bergamot juice

BJ was obtained by hand-squeezing fruits of *Citrus bergamia* Risso et Poiteau collected in the Calabrian region (Southern Italy). Prof. Antonio Rapisarda (pharmaceutical botany expert, University of Messina) has provided the identification of *Citrus bergamia* fruits used to obtain the bergamot juice employed in this experimental study. A voucher specimen of the plant, identified according to the botanical literature, was deposited in the herbarium (H.N. 3263–3266 # CBSD) of the Dipartimento di Scienze del Farmaco e Prodotti per la Salute of University of Messina (Italy).

Aliquots of BJ were stored at −20 °C until further use. The flavonoid composition of BJ was evaluated by RP-HPLC as previously reported [16]. The major flavonoids identified in BJ were eriocitrin, neoeriocitrin, naringin and neohesperidin, of which the most abundant compound was neoeriocitrin (29 % of total), followed by neohesperidin (27 % of total). No significant differences in amounts of eriocitrin and naringin were detected. Minor identified compounds included a number of glucosides, such as apigenin-6,8-di-C-glucoside, diosmetin-6,8-di-C-glucoside and crisoeriol-7-*O*-neohesperidoside-49-glucoside.

### Patients, *H. pylori* strains and culture conditions

Two reference American Type Culture Collection strains of *Helicobacter pylori* (ATCC 43504 and ATCC 49503) and thirty two clinical isolates recovered from gastric biopsy samples of dyspeptic adults (23 women, 9 men; average age, 51 years) undergoing digestive endoscopy at the Endoscopy Unit of the Department of Internal Medicine of the University of Messina, Messina, Italy, were used in this study [14]. None of the patients had previously undergone eradication therapy. All study subjects gave their informed consent and the study was approved by the local ethical committee (Comitato Etico Scientifico A.O.U. Policlinico "G. Martino", Messina, Italy). Diagnosis of peptic ulcer and non-ulcer dyspepsia (NUD) or gastritis was based on endoscopic examination of the stomach and duodenum. Biopsy samples were taken for each patient for culture. Isolates were derived from patients suffering from gastritis (*n* = 27; 84.37 %), or NUD (*n* = 5; 15.62 %).

Gastric biopsy specimens were placed in the sterile screw-capped tubes containing 0.5 ml sterile saline and transported to the microbiology laboratory within 2 h. Samples were soaked and sowed in selective (Pylori agar, BioMérieux, Florence, Italy) and non-selective (Columbia agar with 7 % horse blood, CB, Oxoid, Milan, Italy) culture media. Cultures were incubated for 7 days at 37 °C under microaerophilic conditions. Grown bacteria were identified as *H. pylori* by typical morphology, Gram staining results and positive reactions to oxidase, catalase, and urease activities. The molecular identification of the tested strains has been previously reported [14].

All strains were harvested by suspension in Brucella broth (Becton Dickinson Italia, Milan, Italy) supplemented with 10 % fetal bovine serum (BB, Euroclone, Milan, Italy) and 30 % glycerol and stored in liquid nitrogen until used.

### Susceptibility studies

The minimum inhibitory concentration (MIC) of BJ, amoxicillin (AMX, Sigma, Milan, Italy), clarithromycin (CLA, Sigma, Milan, Italy) and metronidazole (MTZ, Sigma, Milan,Italy) was assayed by the standard agar dilution method according to the guidelines of the National Committee for Clinical Laboratory Standards [25] using CB. Frozen stock cultures were thawed and subcultured on CB and grown for 3 days under microaerophilic conditions. Bacterial growth was taken from the plates, resuspended in BB and grown under shaking (125 rpm) at 37 °C for 24 h. *H. pylori* cultures in the exponential phase of growth were diluted with BB to contain about 5 × 10^7^ CFU/ml by adjusting the turbidity of the suspension to match the MacFarland no. 1 standard. Ten-microliter aliquots of the suspensions were inoculated on CB containing twofold serial dilutions of the compound tested, ranging from 80 (%, v/v) to 0.156 (%, v/v). Compound-free CB media were included in each experiment to confirm the viability of the inoculum and to observe the growth of any contaminants. All plates were incubated at 37 °C in a microaerophilic atmosphere and examined after 3 days. The MIC was considered the lowest concentration at which the compound inhibited the development of visible bacterial growth on the agar plates.

### Combination assays

In the combination assays, the “checkerboard” procedure described by White et al. [26] was followed to determine the efficacy of the association BJ with the three reference antibiotics against all tested strains. The test compounds represented by each antibiotic (AMX, CLA or MTZ) was serially diluted on the x axis, ranging from 1 to 1/64 x MIC, with increasing concentrations of BJ ranging from 1/32 to 1 x MIC on the y axis.

MIC data were converted into fractional inhibitory concentration (FIC), defined as ratio of the concentration of the antimicrobial at an inhibitory concentration with a second compound to the concentration of the antimicrobial by itself as previously reported [27].

FIC_A_ = MIC of A with B/MIC of A

The FIC index was then calculated as follows: FIC index = FIC_A_ + FIC_B_.

All MIC determinations were performed in duplicate for each strain.

### Time-kill curves

Killing curves were performed on the two *cag*A-positive ATCC strains of *H. pylori* (ATCC 43504 and ATCC 49503), on the clinical isolate *cag*A-positive HP6 strain of *H. pylori* and on the clinical isolate *cag*A-negative HP61 strain of *H. pylori*. Tubes containing BJ at concentrations corresponding to 0.5, 1, 2, 4 and 8 x MIC were inoculated with a suspension of each test strain, yielding to a final bacterial density of 5 × 10^6^ cfu/ml and then incubated at 37 °C under microaerophilic conditions (GENbag microaer for microaerophilic bacteria, Bio*Merieux*). A growth control was also performed. Samples for viable counting were withdrawn at 0, 2, 4, 6, 8, and 24 h. At least four dilutions of each sample were spread in triplicate on Columbia agar plates containing horse blood (7 %, Oxoid), incubated at 37 °C under microaerohpilic conditions and counted after 3 days.

Time-kill studies were also performed using a combination of each antibiotic and BJ: solutions of the two drugs were added to tubes containing a suspension of single test strains giving a final bacterial concentration of 5 × 10^6^ cfu/ml. The final concentrations of drugs in each tube were the following: (1) control (without drug); (2) BJ (1 x MIC); (3) AMX (1 x MIC); (4) CLA (1 x MIC); (5) MTZ (1 x MIC); (6) BJ (1 x MIC) and AMX (1 x MIC); (7) BJ (1 x MIC) and CLA (1 x MIC); (8) BJ (1 x MIC) and MTZ (1 x MIC). All experiments were performed in triplicate for each strain.

## Results

### Minimum inhibitory concentrations

The MICs of BJ, AMX, CLA and MTZ against the three reference strains and the clinical isolates of *H. pylori* are reported in Table 1. Results of negative controls indicated the complete absence of inhibition of all the *H. pylori* strains tested (data not shown). BJ (2.5 %) inhibited the growth of 50 % of the clinical isolates tested, whereas 5 % (v/v) inhibited 90 %. AMX was the most effective antibiotic against the reference strains and the clinical isolates, followed by CLA and MTZ. In the combination assays, the lowest FIC index calculated against the reference strains of *H. pylori* was 0.187, which was obtained by the combination of BJ with MTZ (Table 2). The same combination produced the lowest FICI 50 and FICI 90 against the 32 clinical isolates of *H. pylori* (Table 2). Although the interpretation of the FIC indices depends on which of the several definitions described in the literature are used, in this study we have considered as synergistic if the FIC index is </=0.5, additive or indifferent if > 0.5 but </= 4 and antagonistic if > 4. Synergism was observed between BJ and AMX and between BJ and MTZ against both the reference strains and the clinical isolates of *H. pylori*. Indifference was observed between BJ and CLA for all the combinations tested.

**Table 1:** MICs of bergamot juice (% v/v) and reference antibiotics (amoxicillin, clarithromycin and metronidazole expressed as μg/ml) against ATCC strains and 32 clinical isolates of *H. pylori*

	ATCC		CI		
Compound	43504	49503	MIC50	MIC90	MIC range
BJ	2.5	5.0	2.5	5.0	0.625-5.0
AMX	0.0312	0.0156	0.0312	0.0625	0.0156-0.125
CLA	0.0625	0.0312	0.0625	0.125	0.0312-0.125
MTZ	64.0	8.0	2.0	4.0	1.0-16.0

*MICs* minimal inhibitory concentrations, *BJ* bergamot juice, *AMX* amoxicillin, *CLA* clarithromycin, *MTZ* metronidazole, *CI* clinical isolates of *H. pylori*

**Table 2:** FIC index of the association bergamot juice with reference antibiotics (amoxicillin, clarithromycin and metronidazole) against ATCC strains and 32 clinical isolates of *H. pylori*

	ATCC		CI		
Association	43504	49503	FICI 50	FICI 90	FICI range
BJ/AMX	0.375	0.375	0.187	0.308	0.093-0.49
BJ/ CLA	1	0.75	0.75	1.06	0.56-1.24
BJ/ MTZ	0.501	0.187	0.093	0.192	0.093-0.75

*BJ* bergamot juice, *AMX* amoxicillin, *CLA* clarithromycin, *MTZ* metronidazole, *CI* clinical isolates of *H. pylori*

### Time-kill curves

**Fig. 1:**
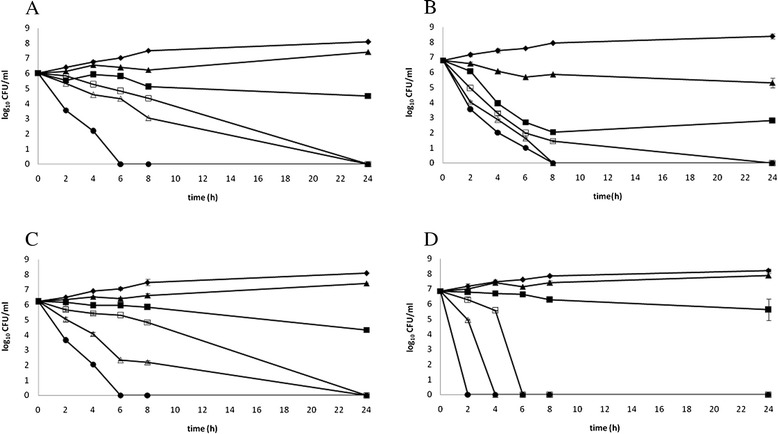
**a**. Killing curves for bergamot juice (BJ) against *H. pylori* ATCC 43504. ▲, 1.25 %; ■, 2.50 %; □, 5.0 %; ▲, 10.0 %; ●, 20.0 %; ♦, control. **b**. Killing curves for bergamot juice (BJ) against *H. pylori* ATCC 49503. ▲, 1.25 %; ■, 2.50 %; □, 5.0 %; ▲, 10.0 %; ●, 20.0 %; ♦, control. **c**. Killing curves for bergamot juice (BJ) against *H. pylori* HP6 (*cag*A-positive). ▲, 1.25 %; ■, 2.50 %; □, 5.0 %; ▲, 10.0 %; ●, 20.0 %; ♦, control. **d**. Killing curves for bergamot juice (BJ) against *H. pylori* HP61 (*cag*A-negative). ▲, 1.25 %; ■, 2.50 %; □, 5.0 %; ▲, 10.0 %; ●, 20.0 %; ♦, control

Concentration dependent killing was observed with BJ against all the strains tested (Fig. 1 a, b, c and d). At 2 h there was a > 3log10 difference in CFU between BJ (1.25 %) and BJ (20.0 %) with *H. pylori* ATCC 49503 (Fig. 1b) and complete bacterial killing was achieved within 8 h exposure at BJ (20 % and 10 %) and within 24 h exposure at BJ (5 %). BJ gave overall less killing against *H. pylori* ATCC 43504, although a complete bacterial killing was achieved within 6 h exposure at BJ (20 %, Fig. 1a). This trend could be explained by the increased resistance to reference antibiotics of *H. pylori* ATCC 43504 compared to *H. pylori* ATCC 49503 (Table 1). The killing curves of BJ against the clinical isolate HP6 (*cag*A-positive) and the clinical isolate HP61 (*cag*A-negative) have shown HP61 was overall more sensitive than HP6 (Fig. 1c and d): a very rapid bactericidal effect was obtained with BJ (20 %) against HP61 after 2 h exposure, whereas complete bacterial killing was achieved with BJ (20 %) against HP6 after 6 h exposure. These results suggest the genotype status of the clinical strains of *H. pylori* may have an impact on their susceptibility to natural antimicrobial compounds.

**Fig. 2:**
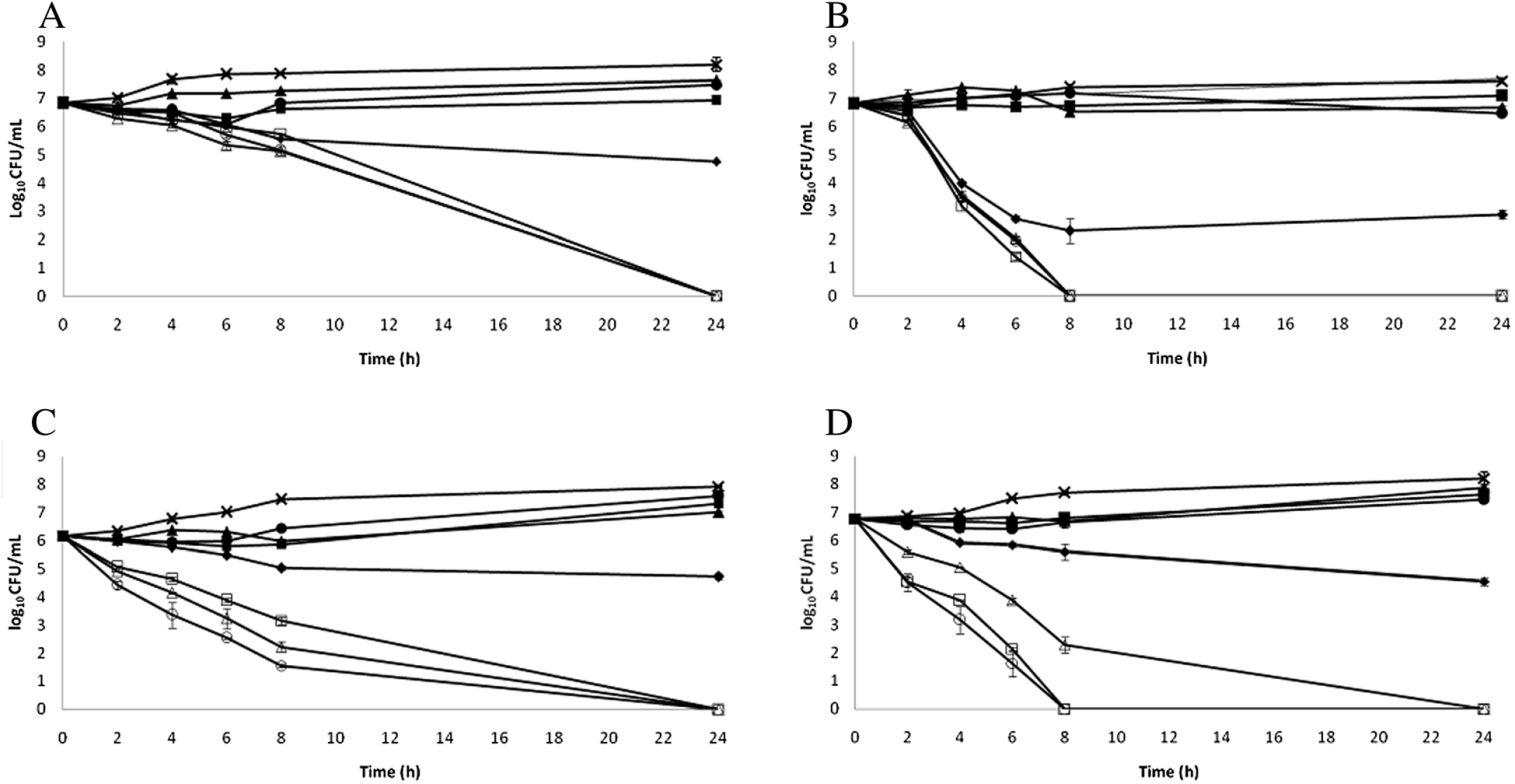
**a**. Killing curves for bergamot juice (BJ) with reference antibiotics (amoxicillin, AMX, metronidazole, MTZ, clarithromycin, CLA) against *H. pylori* ATCC 43504. ♦, BJ (1 x MIC); ■, AMX (1 x MIC); ▲, MTZ (1 x MIC); ●, CLA (1 x MIC); □, BJ (1 x MIC) with AMX (1 x MIC);▲, BJ (1 x MIC) with MTZ (1 x MIC);○, BJ (1 x MIC) with CLA (1 x MIC);×, control. **b**. Killing curves for bergamot juice (BJ) with reference antibiotics (amoxicillin, AMX, metronidazole, MTZ, clarithromycin, CLA) against *H. pylori* ATCC 49503. ♦, BJ (1 x MIC); ■, AMX (1 x MIC); ▲, MTZ (1 x MIC); ●, CLA (1 x MIC); □, BJ (1 x MIC) with AMX (1 x MIC); ▲, BJ (1 x MIC) with MTZ (1 x MIC); ○, BJ (1 x MIC) with CLA (1 x MIC); ×, control. **c**. Killing curves for bergamot juice (BJ) with reference antibiotics (amoxicillin, AMX, metronidazole, MTZ, clarithromycin, CLA) against *H. pylori* HP6 (*cag*A-positive). ♦, BJ (1 x MIC); ■, AMX (1 x MIC); ▲, MTZ (1 x MIC); ●, CLA (1 x MIC); □, BJ (1 x MIC) with AMX (1 x MIC); ▲, BJ (1 x MIC) with MTZ (1 x MIC); ○, BJ (1 x MIC) with CLA (1 x MIC); ×, control. **d**. Killing curves for bergamot juice (BJ) with reference antibiotics (amoxicillin, AMX, metronidazole, MTZ, clarithromycin, CLA) against *H. pylori* HP61 (*cag*A-negative). ♦, BJ (1 x MIC); ■, AMX (1 x MIC); ▲, MTZ (1 x MIC); ●, CLA (1 x MIC); □, BJ (1 x MIC) with AMX (1 x MIC); ▲, BJ (1 x MIC) with MTZ (1 x MIC); ○, BJ (1 x MIC) with CLA (1 x MIC); ×, control

In the combination assays, the association of BJ (1 x MIC) with the reference antibiotics (AMX, 1 x MIC, CLA, 1 x MIC, and MTZ, 1 x MIC) determined a reduction of > 6log10 for *H. pylori* ATCC 49503 after 8 h incubation time (Fig. 2b) and for *H. pylori* ATCC 43504 after 24 h incubation time (Fig. 2a). Similarly to the killing trends obtained with BJ alone, the association of BJ with the three reference antibiotics was more effective against *H. pylori* ATCC 49503 than *H. pylori* ATCC 43504. In the association killing curve a synergistic effect was also observed when treating the clinical isolate HP6 (Fig. 2c) and the clinical isolate HP61 (Fig. 2d). The most effective combination was BJ (1 x MIC) and CLA (1 x MIC) against both clinical isolates, with more rapid bacterial killing achieved with the *cag*A-positive strain (HP6). This data confirmed the combination between BJ and the three reference antibiotics had a synergistic effect against *H. pylori* (both ATCC and clinical isolates). The effect of the association was irrespective of the genotype status of the tested strains.

## Discussion

The results reported in the present study demonstrated for the first time that BJ was effective against *H. pylori* strains, both alone or in combination with antibiotics. We have recently shown that almond skins were active against the same strains with different virulence irrespective of the *cag*A and *vac*A status [14]. In the present study, the genotype status of the clinical isolates affected the suppression of growth exerted by BJ, the *cag*A-negative strain being more sensitive than the *cag*A-positive. The three reference antibiotics (AMX, MTZ and CLA) generally used for the treatment of *H. pylori* were effective in combination with BJ: the standardization of combination therapies may be useful against the increasing numbers of multiple drug resistant strains. All *H. pylori* tested in this study were susceptible to amoxicillin (MIC_90_ 0.25 μg/ml), two (6 %) out of 32 clinical isolates tested were clarithromycin resistant, one of which isolated from a patient suffering from gastritis harbouring the *cag*A+/*vac*As1/m1 genotype, four (12 %) out of 32 clinical isolates tested were resistant to metronidazole.

We have shown that the interactions between BJ and the reference antibiotics (CLA, MTZ and AMX) can improve the effectiveness of the synthetic antibiotic against *H. pylori*. Although the exact mechanism of action is not known, we believe that the synergistic effect may be due to the initial damage of the microbial lipid membrane by the plant compound, which would increase the permeability of the bacteria to the antibiotic. Trombetta et al. [28] have demonstrated that the damage of the microbial lipid membrane function by essential oils could be related to the lipid composition and the net surface charge of the membrane, the Gram-negative bacteria presenting a strong negative charge conferred by the lipopolysaccharide. Other reports on the mechanisms of action of isoflavones and chalcones have indicated an inhibition of the urease secreted by *H. pylori* which is crucial for its survival under the acidic conditions of the human stomach [29, 30]. Several flavone derivatives and other polyphenols in plants are able to inhibit ion and urea conduction and cell vacuolation by VacA, major virulence factor of *H. pylori* [31]. The anti-adhesive properties of BJ could also be responsible for its activity against *H. pylori* [16, 17].

The indifferent effect observed by the combination of BJ with CLA against *H.pylori* could be due to a number of mechanisms, such as inhibition of antibiotic uptake by the bacterial cells compared to MTZ and AMX. Alternatively, direct interaction between the two compounds may result in the reduction of the inhibitory effect. However, in the time-kill studies, the most effective combination was BJ and CLA against both clinical isolates and a synergistic interaction was detected against the two ATCC strains tested.

We have previously demonstrated that polyphenols present in bergamot peel were found to be active against a range of Gram-negative bacteria including *Escherichia coli*, *Pseudomonas putida* and *Salmonella enterica*, and their antimicrobial potency increased after enzymatic deglycosylation [32]. The most representative polyphenols present in bergamot peel were neohesperidin and hesperetin (aglycone), neoeriocitrin and eriodictyol (aglycone), naringin and naringenin (aglycone). We believe that the same compounds present in BJ could be responsible for the antimicrobial effect against *H. pylori* strains [3–7].

## Conclusion

The results of the present study demonstrated that BJ was effective against *H. pylori* strains *in vitro,* both alone and in combination with antibiotics, and could therefore be used as novel strategy for the treatment of antibiotic resistance. Further studies on the mechanism of action of bergamot juice and its interaction with synthetic antibiotics are warranted.

## Abbreviations

*H. pylori*: *Helicobacter pylori*
*cag*A: Cytotoxin-associated gene
*vac*A: Vacuolating cytotoxin gene
PAI: Pathogenicity island
PPI: Proton pump inhibitor
AMX: Amoxicillin
CLA: Clarithromycin
MTZ: Metronidazole
BJe: Flavonoid fraction of bergamot juice
MIC: Minimum inhibitory concentration
FIC: Fractional inhibitory concentration
